# A Comparison of Epitope Repertoires Associated with Myasthenia Gravis in Humans and Nonhuman Hosts

**DOI:** 10.1155/2012/403915

**Published:** 2012-12-02

**Authors:** Kerrie Vaughan, Yohan Kim, Alessandro Sette

**Affiliations:** Immune Epitope Database (IEDB), La Jolla Institute for Allergy and Immunology, La Jolla, CA 92037, USA

## Abstract

Here we analyzed the molecular targets associated with myasthenia gravis (MG) immune responses, enabled by an immune epitope database (IEDB) inventory of approximately 600 MG-related epitopes derived from 175 references. The vast majority of epitopes were derived from the **α**-subunit of human AChR suggesting that other MG-associated autoantigens should be investigated further. Human **α**-AChR was mostly characterized in humans, whereas reactivity primarily to *T. californica* AChR was examined in animal models. While the fine specificity of T-cell response was similar in the two systems, substantial antibody reactivity to the C-terminus was detected in the nonhuman system, but not in humans. Further analysis showed that the reactivity of nonhuman hosts to the C-terminus was eliminated when data were restricted to hosts tested in the context of autoimmune disease (spontaneous or induced), demonstrating that the epitopes recognized in humans and animals were shared when disease was present. Finally, we provided data subsets relevant to particular applications, including those associated with HLA typing or restriction, sets of epitopes recognized by monoclonal antibodies, and epitopes associated with modulation of immunity or disease. In conclusion, this analysis highlights gaps, differences, and similarities in the epitope repertoires of humans and animal models.

## 1. Introduction

Myasthenia gravis (MG) is an antibody-mediated autoimmune disorder effecting neuromuscular transmission. The disease is characterized by episodic muscle weakness and fatigability. MG is a relatively rare disease with an incidence rate of only 200–400 cases per million [1–3]. MG is unique among autoimmune diseases in that the mechanisms of its immunopathology, though not necessarily its etiology, are well characterized [4, 5]. Autoantibodies against acetylcholine receptors (AChR) mediate their depletion at the neuromuscular junction (NMJ), leading to impairment in nerve impulses to the muscles. These anti-AChR antibodies are known to affect neuromuscular transmission by at least three mechanisms: (1) binding and activation of complement, (2) degradation of AChR cross-linked by autoantibodies and (3) functional/physical block of AChR [1]. Cellular responses, in particular class II CD4^+^ helper T-cell activity, is thought to significantly influence and contribute to autoantibody production through cytokines and costimulatory involvement [6–12]. The characterization of both T-cell and antibody epitopes in acquired and experimental MG may help elucidate the complex mechanisms underlying the disease and thereby lead to the development of tolerizing therapeutics where whole-antigen approaches have been problematic [13–17].

The Immune Epitope Database and Analysis Resource (IEDB, http://www.iedb.org/) contains all antibody and T-cell epitope data captured from published literature relating to antibody and T-cell data for human, nonhuman primate, and rodent hosts, as well as a number of other animal species, and encompasses epitopes associated with infectious diseases, autoimmunity, transplantation and allergy (http://www.iedb.org/). As an epitope repository, the IEDB provides a unique resource to inventory and analyze immunological data for a given pathogen or immune-mediated disease. To date, a number of such meta-analyses for human infectious agents, including influenza A, *M. tuberculosis*, Anthrax and Botulinum toxins, Plasmodium parasites, swine flu, and flaviviruses [18–24], as well as allergy [25], have been performed. These analyses provide an overview of the current state of immunological data for a respective disease and highlight specific trends and identify areas in need of further experimentation. Furthermore, these meta-analyses are also meant to increase awareness of the information contained in the IEDB and solicit feedback to further improve the IEDB's usefulness.

The IEDB search interface was designed to provide maximum flexibility and includes the ability to search by the epitope sequence, the epitope type (linear peptide, conformational peptide, or nonpeptidic), epitope source (i.e., AChR), and/or by the genus species of the organism from which the antigen is derived (i.e., human or *Torpedo californica*). Additionally, the user can specify details of the immunological context, including the host species (human, mouse, nonhuman primates, etc.) and the response type (Ab, T cell, MHC binding). Also available in the search interface is the ability to search by the disease status of the host in which immune responses were measured, such as “myasthenia gravis” or “experimental autoimmune myasthenia gravis (EAMG).”

Herein we present an analysis of immune epitope data related to *myasthenia gravis* generated from humans, as well as from animal models of induced MG. Available data from different antigen sources including all AChR subunits are analyzed and pinpointed to their antigen locations. Reactivity is compared for antigens derived from human and nonhuman sources in human and nonhuman hosts. Epitopes specifically associated with disease are also analyzed, as well as those T-cell epitopes for which restriction has been determined, those B cell epitopes recognized by monoclonal antibodies and those antibody and T-cell epitopes associated with disease modulation. This work represents for the first time a comprehensive analysis of immune epitope data for MG and provides us with an opportunity to reach out to the autoimmune community.

## 2. Methods

### 2.1. Targeted Data and Query

All queries were performed using the IEDB home page (http://www.iedb.org/). Queries were specific to antibody and T-cell responses only, MHC binding and MHC ligand elution data were excluded. Unless otherwise specified, results from each query were exported as Excel files and further analyzed in that format to generate particular tables and figures. To identify all records for “myasthenia gravis” or “experimental autoimmune myasthenia gravis (EAMG)” queries were performed using the Disease Finder located on the home page search interface under Immune Mediated Disease Association. The Disease Finder is linked only to those records in which the authors state the presence of disease (past or present) in the patient history. Records captured within the IEDB that do not identify a disease condition will not be included using this feature, but are retrievable through searches specifying antigen and host.

Queries to identify all records defining epitopes for acetylcholine receptor (AChR) were performed using the Molecule Finder under Epitope Source. In order to access all AChRs contained within the database, we made use of the Protein Tree. The Protein Tree is a hierarchical organizer for all epitope source antigens. It is useful for accessing data for which many proteins exist for a given species. Entering “acetylcholine receptor” into the molecule name field specifies the antigen and selecting “human” in the Organism Finder will return all AChRs for this species. Under “Database,” all “PROTREE” designations can be used to bundle all like proteins under a high node. For AChR, a PROTREE identifier is available for all reported AChR subunits (*α*, *β*, *γ*,*δ* and *ε*). Using the higher node on the hierarchical tree allows the user to target all AChRs accessions accumulated for the specified protein. Similarly, the high nodes for *T. californica *AChR and *β*-2-adrenergic receptor were also used. 

Results for all queries were reported in table format, which is similar to what is present on the IEDB Search Results Summary page. If not specified otherwise, the queries return peptidic epitope data for all reported antibody and T cell responses in all hosts (human and nonhuman). The IEDB data are derived from the peer-reviewed literature indexed in PubMed and direct submissions. To be included in the IEDB, epitopes have to be mapped experimentally to a region of 50 residues or smaller. The IEDB captures epitopes and related data as defined in the literature and thus includes minimal/optimal epitopes (8–15 residues), larger less well-defined regions (16–50 residues), and key epitope residues identified as being involved in binding (1-2 residues). The IEDB curation process takes into account the fact that some residues may be important for protein folding instead of binding, and only studies providing controls addressing this issue are curated in the database. Negative structures (defined as structures for which only negative data has been reported) are also captured in the IEDB and have been included in this analysis. Additional detailed curation criteria can be found in [26]. Additional queries were performed to select subsets of MG data using the “T cell search” and “B cell search” functions from the IEDB website, and specifying additional criteria to those mentioned above, such as response phenotype, host organism, organism from which the auto-antigen is derived, or assay type. Figure legends include a summary of query criteria.

### 2.2. Computational Methods—Mapping Response Frequency

The response frequency score (RFscore) is calculated as (responded − square  root(responded))/tested, where “tested” and “responded” correspond to numbers of individuals tested and responded to a given residue. The score has a range [0 to 1], and a higher score indicates that a larger fraction of individuals responded. The square root is a correction factor, approximating one standard deviation for the number of responding donors. This gives a higher score to epitopes studied with larger sample sizes. For reference, an epitope positive in 10/10 donors will yield an RFscore of (10 − square  root(10))/10 = 0.68, and an epitope positive in 100/100 donors tested will have an RFscore of 0.90. This formula can also be applied to structures that were negative in all assays and donors tested. 

Epitopes derived from each autoantigen were mapped to a position in the reference protein sequence (human AChR (GI: 4261947)). To assign a position to a peptide epitope, all peptides were mapped using a 40% threshold of sequence homology, chosen to allow mapping of homologous sequences derived from different species, to each protein and the best matching position was kept. Second, all peptide-matched positions were renumbered with respect to the reference protein. Here the reference antigen differs in position by 20 amino acids. The numbering of synthesized peptides did take into account the first 24 amino acids of the full-length protein. For example, a synthesized peptide called “1–20” corresponds to residues 25–44 (starting position is 25, not 1) on the full-length alpha subunit of ACHR. 

## 3. Results

### 3.1. A High-Level Inventory of MG Immune Epitope Data

**Figure 1:**
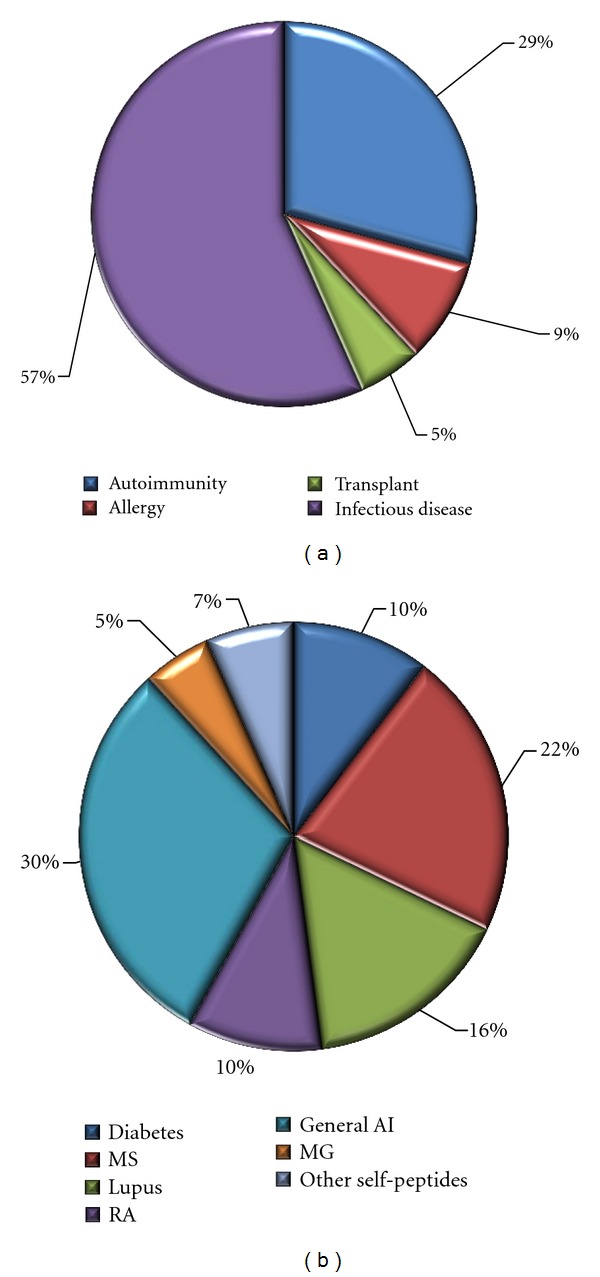
(a) Relative abundance of references related to different disease categories. (b) Subcategorization of autoimmunity references.

Previous studies [27] have described a broad scheme for classification of immune epitope references, and subsequent work has described the development of an automatic classification process that is utilized in conjunction with human expert review [28]. This schema was utilized to identify the references analyzed in the current study. As of April 2012, the IEDB contains >3,800 references categorized as autoimmune (AI) disease Related. This represents nearly 30% of all the reports captured in the database (Figure 1(a)), second in abundance only to infectious diseases. The AI category is further organized into 7 subcategories, including diabetes, lupus, multiple sclerosis, rheumatoid arthritis, and myasthenia gravis.  Figure 1(b) shows the relative proportion of the each subcategory within the AI category. 

A total of 175 references associated with nearly 600 epitopes (structures associated with at least one positive assay) are related to *myasthenia gravis,* which represent 5% of the “Autoimmune” category. This number of references matches the number of references that can be retrieved by entering “myasthenia gravis” in the keyword search field of the IEDB main page. Thus, a relatively large number of references relating to MG and its associated antigens are available to the scientific community. The analysis of the data contained in these references is the object of the present paper.

### 3.2. Narrow Focus of Antigenic Sources for MG Data

MG-related epitopes are in large majority derived from AChR.  Table 1(a) shows the result of individual queries performed to show all data for AChR by species. The majority of epitopes was defined for human AChR (338), followed by *Torpedo californica* (Pacific electric ray, *T. californica*) (175) and a smaller number from chicken, rat and bovine AChR (21, 16 and 11, resp.). Very few epitopes have been defined for *Torpedo marmorata* and mouse AChR (6 and 5, resp.).

Epitopes for one additional MG-related antigen were also reported (Table 1(b)). Beta-2-adenergic receptor (*β*2-AR) is considered to be an MG autoantigen; up to 25% of MG patients have anti-*β*2-AR and *β*1-AR antibodies [29]. Moreover, MG patients have been shown to have humoral and cellular responses to *β*2-AR peptides [30, 31], and less than normal density of such receptors on peripheral blood mononuclear cells [32]. Epitopes were not described for any additional MG-related antigens. 

**Figure 2:**
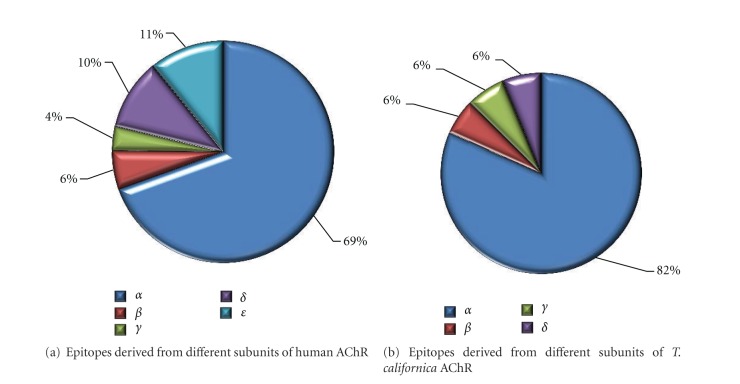
Data were generated by querying for all (a) human AChR proteins or all (b) *T. californica* AChR proteins in the Molecule Finder and then exporting the positive assay data in Excel format. The percentage of epitopes derived from each subunit was then determined by sorting by “Source Molecule Name” field for each subunit designation.

Next, in the case of AChR, we investigated the representation of different subunits as the source of the described epitopes. The vast majority of epitopes were derived from the alpha subunit of *T. californica* and human AChR, though limited data were also available for the beta, gamma, delta and epsilon subunits (Figures 2(a) and 2(b)). A unique feature of the IEDB is not only the positive data that is curated, but also experiments in which a given peptide was tested and found to be negative are curated as well. Further analyses were performed by taking advantage of this feature, and taking into consideration the ratio of positive and negative records (Table 2). The results suggest that in comparison to the alpha subunit, the delta and epsilon subunits are also fairly well studied, and are associated with mostly negative data, whereas the beta and gamma subunits are under investigated. 

### 3.3. Types of MG-Associated Epitopes Defined in Different Hosts

As mentioned above, approximately 600 unique MG associated epitopes (structures associated with at least one positive assay) are described within the IEDB. Of the 1,891 assays curated, 556 were defined in the context of B cell/antibody assays, and 1,335 in the context of T-cell assays. These totals include linear and nonlinear epitopes, as well as mimotopes/analogs, mainly derived from human or *T. californica* acetylcholine receptor (AChR). Surprisingly, only 5 conformational B cell epitopes have been reported to date from either human or *T. californica* AChR and only two of these were defined in humans (data not shown). For T cell responses, CD4^+^ T cells are most relevant as they are critical in the generation of the auto-antibodies that are responsible for the MG-related pathology. Accordingly, most studies focused on CD4^+^/class II restricted T cells (~82%), and for most of the remaining epitopes the phenotype of the responding T cells was undetermined and therefore not assigned as either CD4^+^ or CD8^+^. Few if any class I epitopes have been reported to date. 

**Figure 3:**
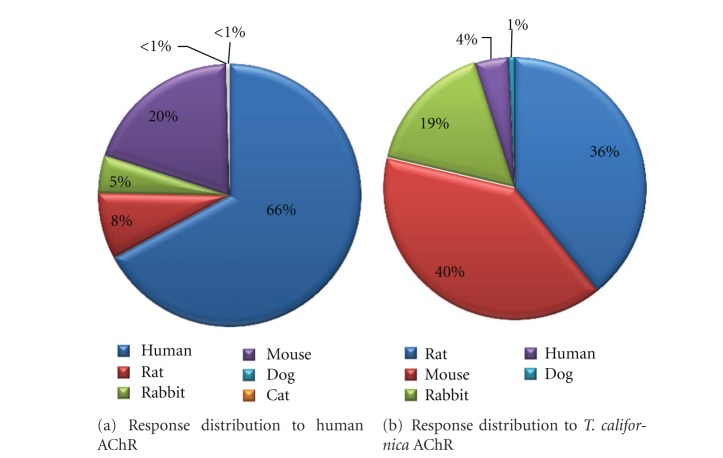
Queries utilized the Source Antigen Molecule Finder to specify AChR, selecting B cell and T cell responses only (MHC binding and MHC ligand elution assays were excluded), and specifying Host Organism. The total number of positive epitopes was then tallied to generate relative percentages of reactivities.

Immune responses have been characterized in a variety of different hosts, including humans, dogs, cats, rabbits, rats, and mice (including human HLA-transgenic mice). Figures 3(a) and 3(b) show the proportion of host data for human and *T. californica *AChRs. The vast majority of reactivity to human AChR was characterized in human subjects, whereas the bulk of reactivity to *T. californica* AChR was described in rats or mice. This system has been historically utilized to study AChR reactivity, since this fish represented a convenient and abundant source for biochemical isolation. Tables 3(a) and 3(b) provide a detailed breakdown of these responses per epitope. T-cell data for human AChR is relatively more abundant than B cell/antibody data. In general, reactivity measured against *T. californica* AChR has been primarily focused on antibody responses, which outnumber T-cell responses by nearly 3 to 1. Finally, Table 3(c) shows responses against all other AChRs. Here, the vast majority of epitopes were defined in the context of antibody responses using rats and rabbits. The focus of humoral responses in rabbits is likely historical, as it was work performed in rabbits that first defined the autoimmune nature of MG [33–35]. 

### 3.4. Differences and Similarity in Immune Reactivity to AChR Derived from Humans and Torpedo Californica

Most MG research utilizes the alpha subunit of AChR derived from humans, as well as *T. californica*, which is used as a convenient source of AChR in animal studies. Sequence homology between the two antigens for the extracellular domain is only 87%, whereas the C-terminal regions are 94% homologous. The N-terminal region represents the extracellular portion of AChR and antibodies directed against this region are likely pathogenic because they engage the receptor binding site or its vicinity. Indeed, the extracellular domain is well known for its immunogenicity, attributable almost entirely to the main immunogenic region (MIR) [36, 37]. The MIR is located on the *α*1 loop of AChR (aa66–76) [38] and is the target of more than half of all MG patient auto-antibodies [39, 40]. Even at this well-established site, the human sequence [41] varies from that of *T. californica*. Similarly, the major antigenic region (MAR, aa125–147), differs from that of Torpedo AChR [38]. Thus, despite the high level of overall sequence homology between the human and Torpedo AChRs, existing sequence variations could lead to differences at the level of B and T-cell reactivity. 

We recently developed an approach to map individual query results onto a specified reference genome or antigen [42]. This immunobrowser tool provides a response frequency score (RFscore), reflecting the overall frequency by which epitope structures containing a given residue are assayed and recognized. Here, the immunobrowser was used to map antibody and T-cell epitope reactivity from human and nonhuman hosts, onto a reference AChR sequence (GI: 4261947). 

The majority of the data from human hosts relate to reactivity to the human self-antigen. By contrast, the majority of nonhuman data are derived from the homologous but non-self-antigen, Torpedo AChR. Therefore, we wanted to investigate the extent to which immune responses defined for each host overlapped or were distinct. To do this analysis, we compared responses from human subjects to human AChR (self) to responses of nonhuman (rats, mice, and rabbits) hosts to all non-self-AChRs (which included Torpedo and human antigens). Sequence homology among the alpha subunits of mammalian AChRs (human, rat, mouse, rabbit, etc.) is 90% or higher. A comparison of response frequency of non-human hosts to their self AChRs was preformed; however, the data were too few to allow meaningful analysis, highlighting the fact that even in studies defining reactivity induced by the non-self-Torpedo, definition of epitopes to self antigen is not widely done.

**Figure 4:**
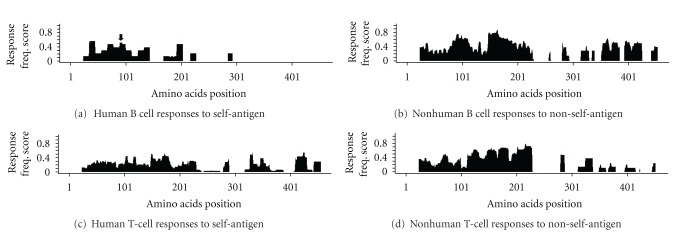
Response frequency response.

Figure 4(a) shows response frequencies of epitopes derived from human AChR for antibody reported for human subjects. Human antibody responses to human AChR are focused on the N-terminal extracellular domain, specifically residues ~20–150 and 160–200, which include well-known epitopes MIR aa66–77 (here aa86–97, black arrow), which is provided as a point of reference. A small gap in reactivity was observed between residues 150–160. In the case of nonhuman hosts, B cell responses also recognize the extracellular domain (Figure 4(b)). However, frequent responses are observed in the C-terminal domain, especially between residues ~280–450, which were not reported to human reactivity to self-antigen. Furthermore, more frequent responses to the 150–220 were observed in animal studies to non-self AChR than in human anti-self-AChR responses. Human reactivity to non-self-epitopes and reactivity of nonhuman hosts to self-molecules were sporadic and too sparse to allow for a meaningful analysis (data not shown).

T-cell responses were analyzed next. Human T-cell responses to self-antigen show a broad reactivity along the most of the AChR protein, with concentrated responses between residues ~20–220, 320–370, and 410–450, this includes an RFscore of 0.64 at aa166-182, containing the well-known class II epitope (~146–162) [14], (Figure 4(c)). Nonhuman T-cell responses to non-self-antigen, which includes Torpedo and human ACHR, were similar to those of human to self antigen, but with differences in the magnitude of RFscores; T-cell frequency was higher in the N-terminal extracellular domain for nonhuman hosts, whereas RFscores observed in the C-terminal domain were slightly higher in human subjects. (Figure 4(d)). In summary, while the fine specificity of T-cell response was similar in the two systems, the epitope targeted by antibody responses were markedly different, with substantial reactivity to the C-terminus of alpha-AChR detected in the nonhuman system, but not in humans.

### 3.5. Are Negative Regions Truly Negative or Simply Untested?

Next, we wanted to further investigate the extent to which the “sporadic reactivity” and “response gaps” observed in the response frequency data presented above correlated with regions that were untested versus those that had been tested and were found to be negative. This would provide insight into which regions of AChR were truly nonimmunogenic versus those regions that may have been under investigated to date. 

Using the negative data captured in the IEDB, the total number of negative assays per residue was plotted along the reference antigen (data not shown). Overall, we found that response gaps did indeed represent untested regions for the vast majority of scenarios presented above. There were, however, three notable exceptions. In the case of T-cell responses to self-antigen in human subjects 100% of the antigen is represented; with all response gaps representing negative regions. Interestingly, the same is not true for human antibody reactivity to self-antigen. Here, we see that only 40% of AChR represents positive data and the remainder of the antigen is untested. In the case of non-human T cell responses to non-self, 98% of the antigen has been tested, with only a small region of untested residues (aa383-393). Nonhuman antibody responses to non-self represent 88% of the antigen, with four small, untested regions in the C-terminus. Finally, the first 20–24 residues (signal sequence) were not represented in the epitope data, as most overlapping peptide studies began with residue 25.

### 3.6. Analyzing the Epitope Data Associated with Clinical and Experimental Disease

Next, we hypothesized that the differences detected in the human versus nonhuman system might reflect differences in the disease state associated with the human and nonhuman hosts. To test this hypothesis, we further investigated how closely the epitope specificity observed in animal models of disease would mirror that observed in human patients. This type of analysis requires being able to select only records clearly associated with clinical or experimental disease. 

The Disease Finder is a recent feature of the IEDB interface that allows querying specifically by host disease status, as reported in each study. Relevant to this analysis, a designation of “myasthenia gravis” includes all records of human MG patients, as well as those from dogs and cats (also prone to acquired MG), and “experimental autoimmune myasthenia gravis (EAMG),” which includes those records associated with rat, mouse and rabbit data. Studies that do not specify disease status, but are related to MG (e.g., studies involved in the generation of monoclonal antibodies by immunization with AChR), will not be found using the Disease Finder, but are retrievable through a query specifying a specified AChR and host.

Using the Disease Finder to query for MG we find a total of 301 positive epitopes (unique molecular structure) from 49 references (Table 4). These epitopes are derived from human and *T. californica* acetylcholine receptor (AChR), human beta 2-adrenergic receptor (ADRB2), as well as mouse, bovine, rat and *T. marmorata* AChR. The vast majority of epitopes have been defined in humans with MG, though data also exist for dogs and cats. A second query for EAMG yielded 78 epitopes reported from 39 references. These epitopes were derived from human, *T. californica* and rat, AChR. Animal models included rabbits, Lewis rats, and mice (including HLA transgenic mice).

**Figure 5:**
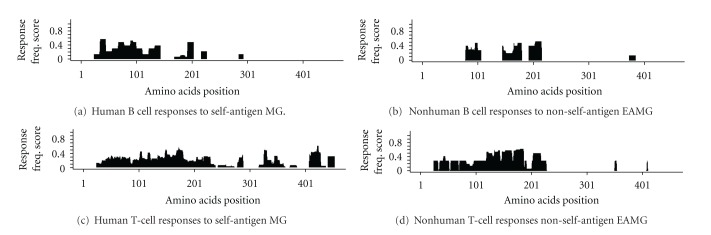
Response frequency of Antibody response for clinical or induced disease.

Applying the disease criterion to the Immunobrowser, we then compared epitope specificity defined in animal models of EAMG with that observed in humans with MG. For antibody reactivity, both the human self-reactivity and the nonhuman host non-self-reactivity were distributed across most of the N-terminal extracellular domain and were lacking in the C-terminus (Figures 5(a) and 5(b)). Thus the additional reactivity detected in the case of nonhuman hosts against non-self AChR in the C-terminal region, is essentially eliminated when the data are restricted to hosts tested in the context of autoimmune disease. In total, adding the criterion of disease to the analysis of nonhumans against non-self AChR eliminated 60% and 87% of the records plotted in Figure 4 for T cell and B cell reactivity, respectively. Conversely, the pattern observed for T cell reactivity to non-self antigen in animals with autoimmune disease differed from responses of human MG patients to self-antigen (Figures 5(c) and 5(d)). Here we see a lack of T cell reactivity in the C-terminus in EAMG models. This was unexpected, since a reasonable correspondence had been observed previously without removing records not clearly associated with MG disease. The reason for this difference is unclear; however, it may reflect the fact that in animal models, EAMG is induced by cross-reactivity to self-AChR.

### 3.7. HLA Association and Susceptibility to Disease

**Figure 6:**
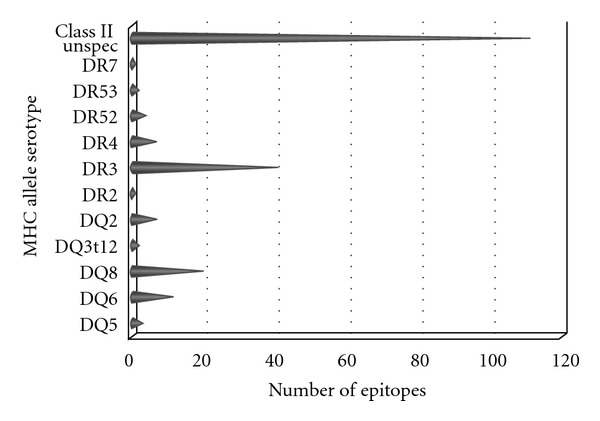
Restricting MHC allele. Query included human AChR as antigen, T-cell responses only (B cell responses, MHC binding, and MHC ligand elution assays excluded) and MHC class II selected. Enumeration of each allele was done using Excel download of positive T-cell responses. Data include humans, as well as HLA-transgenic mice as host.

At the genetic level, susceptibility to MG is most frequently associated with the class II alleles DR3, DQ8, and DQ6 [43–45] in humans and Ia in mice [46, 47].  Figure 6 shows the overall profile of MHC restriction demonstrated *in vitro* (by assay). HLA-DR3, DQ6, and DQ8 are fairly well represented with much smaller numbers of epitopes defined for the other serotypes. Interestingly, though DR3 represents the largest number, all but 2 epitopes were generated using HLA-transgenic mice. In fact, of all the data with defined restriction, only 36% was related to human subjects. 

As noted above, in the case of human data, definitive restriction data is often not available. However, a number of studies inferred restriction on the basis of the HLA types of the subject in which a positive response was detected. This type of data is also curated in the IEDB and can be retrieved using the advance search on the home page. By these criteria, we find a number of additional epitopes defined in humans of known susceptible HLA serotypes, namely DQ and DR alleles (Table 5). Thus, the curation strategies of the IEDB preserve its granularity of the data, and provide a significant array of well-defined epitopes for use by the scientific community.

### 3.8. Querying for Epitopes of Particular Biological Relevance

The IEDB allows selecting epitope sets of particular interest based on the experimental details associated with each epitope. Here we discuss two examples, namely, B cell epitopes bound by well-defined monoclonal antibodies and T-cell epitopes involved in disease modulation.

AChR-specific monoclonal antibodies have been defined and tested extensively in EAMG. These reagents have been shown to induce MG by passive transfer in animal models and have also been applied therapeutically to reduce disease symptoms [48–50].  Table 6 provides a list of monoclonal antibodies reported to date associated with known specificity, and includes the reported epitope-specificity (linear sequence) and indicates whether the mAb has been shown to cross-react with human AChR. Where possible, the reactivity was clustered such that the overall “coverage” of AChR by mAbs could be visualized.

A major objective of epitope research is the identification of epitopes involved in pathogenesis induction, as well as those associated with disease resolution and induction of immune tolerance. While the former peptides are more often used for investigating mechanism of disease, the latter are of interest with respect to immunotherapy.  Table 7 provides a list of all epitopes reported as myasthenogenic, as well as those identified empirically to induce tolerance or to reduce symptoms of EAMG. In several cases we see the same epitope present in both categories. These peptides have been shown to tolerize or exacerbate MG disease depending upon whether administration preceded or followed AChR administration.

## 4. Discussion

Herein we provide for the first time, systematic review of MG-related immune epitope data, including antibody and T-cell epitopes defined for human and *T. californica *AChRs, in humans and in animal models of disease. The purpose of this study was to provide a balanced and comprehensive inventory of all data reported to date, to better understand the data as a whole, as well as to highlight interesting trends and identify knowledge gaps, rather than formulating hypotheses or provide critiques.

The data considered were captured from the published literature and are housed in the IEDB (http://www.iedb. org/), a freely accessible repository of epitope data sponsored by the National Institute of Allergy and Infectious Disease (NIAID). The IEDB was designed to make immunological data easily available to researchers and searchable by multiple experimental parameters, such as sequence, by antigen, by organism, or by disease [26]. The IEDB captures all immune epitope data meeting the criteria established by NIAID (http://www.iedb.org/; curation manual) and contains only those structures empirically defined as binding adaptive immune cell receptors (TCR and BCR); the database does not impose judgment for the purpose of inclusion. The IEDB instead represents an assay-centric repository in which all curated observations are given equal weight, and the relevance of individual data is left up to the user. In this way, the data can be considered as a whole without bias, and/or can be scrutinized to any level of stringency desired.

Not surprisingly, the vast majority of epitopes were defined for human AChR in the context of clinical disease, and CD4^+^ T-cell epitopes were far more numerous than antibody epitopes. This was interesting in the context of the main mechanism of disease, namely, antibody-mediated dysfunction at the neuromuscular junction [7–9]. We also detected a paucity of reported conformational antibody epitopes, despite numerous citations related to the generation and characterization of AChR-specific monoclonal antibodies. Since linear determinants in general account for a minority of epitopes recognizing native proteins [51], the epitope specificity of antibody responses in human MG remains largely incomplete, with B cell epitopes representing less than 10% of the total epitopes defined in humans. Further investigation in this area is therefore warranted.

With respect to species that provided the antigenic source of the epitopes, most epitopes were derived from either human or Torpedo AChR. Torpedo AChR epitopes that were defined mostly in animal models, were predominantly antibody determinants rather than T-cell epitopes. This observation is likely a reflection of the fact that until recently definition of human antibody epitopes has been considerably more challenging than in animal models. The number of epitopes defined for *T. californica *AChR were approximately half that of the human antigen and were mostly studied in rats and mice, but were also tested in rabbits and humans. Data describing bovine, rat and mouse AChR epitopes also existed, but these records were far less numerous by comparison to human and *T. californica *AChRs.

In terms of the antigens from which the epitopes are derived, while all AChR subunits were represented in the epitope data, the main focus was on the alpha subunit, which alone accounted for 69% and 82% of the total response for human AChR and Torpedo AChR, respectively, while the focus on the alpha subunit is not unexpected [40, 48], other subunits have been shown to be involved in disease [52] and therefore may warrant further study at the epitope level. There are also no data related to antigens other than AChR, with the exception of *β*-2-adrenergic receptor for which few epitopes have been reported. When compared to repertoire defined for other autoimmune diseases, such as diabetes, lupus, MS, and rheumatoid arthritis for which numerous self-antigens have been defined, this is exceptional. This observation is also especially interesting in the context of epitope spreading which has been discussed as it relates to MG. Indeed, several other autoantigens have been identified as being involved in patients with early onset or chronic disease [53]. These antigens include muscle-specific kinase (MuSK), which is required for the formation of the neuromuscular junction and represents the second most frequently recognized autoantigen in MG patients [54], as well as antibodies against titin [55] or troponin I [56]. No defined epitopes have been reported for these antigens. 

Here, the use of the Immunobrowser to map the frequency of T- and B-cell responses by residues represents a novel approach to analysis of immune reactivity. A major advantage of this tool is that it allows the user to visualize the regions associated with the highest responses frequencies, and does so by accessing data across different studies, hosts, assays, response types, and so forth. More significantly, this is the first of its kind analysis of autoimmune reactivity to a self-protein, including the analysis of reactivity to exogenous and endogenous antigens among different host species used as models of disease.

Because Torpedo AChR is not a self-antigen, and yet is frequently utilized in the context of experimental MG studies, we thought it would be of interest to compare the epitope repertoire observed in the different host (human/nonhuman) and antigen (self/non-self) combinations. Our analysis revealed that in general reactivity to non-self antigens in humans and conversely self antigens in nonhuman systems is scarce, and in general an apparent lack of reactivity cannot be ascribed to a real lack of reactivity, but more likely is associated with a lack of sufficient studies (to be discussed more below). 

Based on the overall volume of data available, reactivity of human hosts and self-AChR could be meaningfully compared to reactivity of nonhuman hosts to non-self (mostly Torpedo and human). This analysis revealed a general correspondence at the level of T-cell responses. However, in the case of B cell responses, significant reactivity was noted for the C-terminus of the molecule in the case of nonhuman hosts, which was not matched in the case of human subjects. The response difference observed between hosts was much reduced or eliminated when only records clearly associated with MG-diseased hosts were considered. This result suggest that the C-terminus reactivity detected in nonhuman hosts might be nonbiologic in nature and also suggests that selection of nonhuman records most closely matching human disease might be key in immune epitope studies. Overall, the data suggest that the epitope reactivity of Torpedo AChR is a reasonable model of the human AChR reactivity in humans, but only if the disease state of the nonhuman host is also considered. 

Previous meta-analyses [18–25] have targeted epitopes derived from microbial antigens (Plasmodium species, influenza A, mycobacterium, *Bacillus anthracis*, *Clostridium botulinum*, Flavivirus, poxvirus, and HCV) or allergens; this work represents the first analysis of epitopes derived from an autoantigen, in the context of autoimmune disease. Despite the disparate nature of the topics covered to date, one consistent observation was the existence of investigational or experimental bias. This is the phenomenon by which researchers in a particular field of study tend to repeat certain experimental methods, heavily target well-characterized antigens/allergens, and/or ubiquitous pathogen strains/genotypes or allergens, thereby creating a “bias” in the literature that is difficult to distinguish from biological truths.

An example of this is “immunodominance.” In the literature, one may find that, for example, reactivity to the circumsporozoite protein (CSP) from P. falciparum or hemagglutinin (HA) from influenza A may dominate the data in terms of volume, suggesting relative immunodominance. However, it is often the case that these antigens have simply been more heavily investigated than others due to experimental bias and are not necessarily the only antigens of significance in their respective systems. Similarly, overlapping 15–20 mer peptides are used predominantly to define antibody reactivity versus crystallography or other more time-intensive or expensive methods, though most would agree that B cell epitopes are largely nonlinear.

A similar investigational bias was observed for MG-related epitope data. A somewhat provocative and unexpected revelation from these data had to do with the nature of the antigen targeted in animal studies for MG. In humans, acetylcholine receptor is an intrinsic antigen and the target of auto antibodies. However, the study of disease in nonhuman animal models frequently uses an extrinsic (albeit highly homologous) or non-self-AChR (from Torpedo) to induce cross-reactivity to self-AChR in order to mimic clinical disease. The surprising finding was though mechanistically all immunopathology induced in these models would be to the endogenous antigen, and not to the immunizing agent, very few studies mapped epitopes to the self AChR, but rather focused on defining epitopes in Torpedo AChR. In fact, if we use the Immunobrowser to map all mouse T-cell reactivity (host = Mus musculus) to a reference murine AChR only 45% of these are mapped to the endogenous AChR using a peptide similarity threshold of 100%. If the threshold is set to 90% to accommodate the differences between mouse and human AChR (which are 95–98% homologous), still only 75% are mapped, suggesting that one quarter of the data do not map to the endogenous antigen (data not shown). This comparison suggests that there are legitimate differences between the intrinsic and extrinsic AChR that are manifested immunologically and therefore may influence interpretation of animal studies using TAChR.

Overall, the database provides access to data of biological relevance, including monoclonal antibody data, epitopes associated with immunomodulation, as well as those specifically defined in the context of clinical/induced disease. Indeed, of the approximately 500 independent T cell assay records defining human MG reactivity in the IEDB, 25% provide either HLA typing of the subjects tested and/or by restriction of the *in vitro *assay, thus providing the MG community with a catalog of epitopes to use in the most relevant restrictions and those associated with disease. While ample data exist to provide useful analysis, there are important gaps in these data to be noted. Firstly, given the strong HLA association with AI diseases in general and MG in particular, it was surprising that more epitopes of defined restriction were not described. If we consider only those epitope for which MHC restriction was defined for humans in the assay, a mere 18 of the 269 (~7%) unique epitopes are HLA-restricted. Similarly, and as mentioned above, while numerous mAbs have been defined and have been demonstrated to be relevant for diagnostic or immunotherapeutic application, few discontinuous epitopes have been mapped from these. 

Molecular mimicry as an etiology for autoimmune sequelae was not considered in the current study. While this association has been studied by several groups and include pathogens such as *H. influenza*, *M. tuberculosis* [57], Human herpes virus 1 [58], rotavirus, *V. cholera*, New Castles, *S. thyphimurium*, *M. pneumonia*, and others [59], data in the IEDB do not demonstrate *ex vivo* responses of human MG patient sera with whole pathogens or native protein antigens. Rather, only a small number of studies show reactivity of MG sera or antibodies from rodent models to synthesized pathogen-specific peptides or show response through the use of cell lines/epitope-affinity purified effectors reacting to peptides and/or organisms. While this does demonstrate the potential for mimicry at the molecular level, it does not provide evidence for a true causal relationship. In this light, we feel that the data are insufficient to provide further insight.

Herein we have presented a comprehensive analysis of immune epitope data related to myasthenia gravis generated from humans, as well as from animal models disease. Analysis of the overall data has highlighted areas in need of further study, and raised important issues related to the difference in the breadth of epitope repertoires detected in human populations versus animal models of disease. Ultimately, we hope that this work has increased the overall awareness of the IEDB resource by the MG research community and will encourage feedback, a key element to its continued enhancement.

## Figures and Tables

**Table:** (a)

Antigen	Positive	Negative	Total tested	References
Acetylcholine receptor (AChR)				
Human epitopes	**338**	80	418	90
T-cell assays	704	575	1279	
B cell assays	194	71	265	
*T. californica* epitopes	**175**	60	235	102
T-cell assays	495	426	921	
B cell assays	462	53	515	
Chicken epitopes	**21**	16	37	3
T-cell assays	0	0	0	
B cell assays	26	16	42	
Rat epitopes	**16**	0	16	6
T-cell assays	19	0	19	
B cell assays	44	0	44	
Bovine epitopes	**11**	0	11	3
T-cell assays	11	6	17	
B cell assays	5	0	5	
*T. marmorata* epitopes	**6**	0	6	3
T-cell assays	6	0	6	
B cell assays	5	0	5	
Mouse epitopes	**5**	3	8	8
T-cell assays	7	4	11	
B cell assays	12	0	12	

Data was derived from the IEDB Search interface using the Epitope Source Molecule Finder to retrieve epitopes from all AChRs subunits in B cell and T-cell response assays, irrespective of the host in which the immune response was described. Search criteria included “acetylcholine receptor” or “cholinergic receptor” (in the case of rats) for the Molecule Name and the AChR species from the Organism Finder (e.g., *Mus musculus*, human, Torpedo, etc.). In cases where multiple AChRs accession numbers were linked to the various references, all available identifiers were used, including protein tree identifiers (ex. PROTREE (PT10006210)) and Internal identifiers (ex. AChR IEDB (SRC248684)). Listed in the table are the total numbers of epitopes (distinct molecular structure) and then distinct assays captured within the database. By definition this number does not indicate the number of individual epitopes, as each epitope is defined experimentally by one or more assays.

**Table:** (b)

Antigen	Epitopes	Negative peptides	Total tested	References
*β*-2 adrenergic receptor				1
Human	**3**	0	3	
T-cell assays	0	0	0	
B cell assays	5	0	5	

Search criteria included “adrenergic receptor” for the Molecule Name, and each different species for which data was available (as determined from the list of hosts in the keyword search) from the Organism Finder. In the case of adrenergic receptor, though a total of 7 references were found, only 1 (PMID: 1378277) was specific to MG epitopes; the others were related to other autoimmune disease states. Listed in the table are the total numbers of epitopes (distinct molecular structure) and then distinct assays captured within the database. By definition this number does not indicate the number of individual epitopes, as each epitope is defined experimentally by one or more assays.

**Table:** (a)

Subunit	Positive epitopes	Negative peptides	Positive assays	Negative assays	Neg/pos ratio
*α*	235	61	746	513	0.69
*β*	19	0	29	0	0
*γ*	12	0	23	1	0.04
*δ*	35	11	58	82	1.4
*ε*	37	8	42	50	1.2

**Table:** (b)

Subunit	Positive epitopes	Negative peptides	Positive assays	Negative assays	Neg/pos ratio
*α*	143	32	895	439	0.49
*β*	10	0	14	0	0
*γ*	11	0	28	2	0.07
*δ*	11	28	20	38	1.9

Listed in the table are the total numbers of epitopes (distinct molecular structure) and then distinct assays captured within the database. By definition this number does not indicate the number of individual epitopes, as each epitope is defined experimentally by one or more assays.

**Table:** (a)

	B cell epitopes	T-cell epitopes	Total
Host			
Human	20	260	280
Mouse	25	62	87
Rat	29	5	34
Rabbit	13	0	13
Dog	1	0	1
Cat	1	0	1
	**99**	**327**	**416**

**Table:** (b)

	B cell epitopes	T-cell epitopes	Total
Host			
Mouse	62	42	104
Rat	75	28	103
Rabbit	43	0	43
Human	5	4	9
Dog	2	0	2
	**187**	**74**	**261**

**Table:** (c)

	B cell epitopes	T-cell epitopes	Total
Host			
Mouse	4	5	9
Rat	24	1	25
Human	0	11	11
Rabbit	20	0	20
	**48**	**17**	**75**

These data were derived using the Source Antigen Molecule Finder to specify AChR, selecting B cell and T-cell responses only (MHC binding and MHC ligand elution assays were excluded). For this analysis Host Organism was not specified. Positive T-cell and B cell Response data were downloaded separately into Excel format and further analyzed to determine the number of B and T-cell epitopes per host species reacting to either AChR. Listed in the table are the total numbers of epitopes (distinct molecular structure).

**Table 4:** Disease-associated data.

Disease	Epitopes	Negative peptides	Total tested	References
*Myasthenia gravis *	**301**	104	405	49
T-cell assays	**434**	273	707	
B cell assays	**49**	19	68	
*Experimental autoimmune, Myasthenia gravis, EAMG *	**78**	10	88	39
T-cell assays	**152**	83	235	
B cell assays	**62**	12	74	

These data were derived by selecting “myasthenia gravis” as the disease. Host organism was not specified; therefore the data for MG will include dog and cat as hosts, in addition to human subjects. MHC binding and MHC Ligand elution assays were excluded. “Positive epitopes” refer to those peptides/structures found to be positive in at least one measurement. “References” refer to studies published in peer-review literature and contained in PubMed. Acquired MG includes human, cat and dogs as host. EAMG includes rabbits, rats and mice, including human transgenic mouse strains. Listed in the table are the total numbers of epitopes (distinct molecular structure) and then distinct assays captured within the database. By definition this number does not indicate the number of individual epitopes, as each epitope is defined experimentally by one or more assays.

**Table 5:** HLA association.

HLA serotype	Epitope sequence from hAChR
A2, A29, B7, DR2, DR7	STHVMPNWVRKVFIDTIP
	IPNIMFFSTMKRPSREKQ
	AIVKFTKVLLQYTGHITWTP
	QIVTTNVRLKQQWVDYNLKW
	IIGTLAVFAGRLIELNQQG
DQ9, DQ6, DR9, DR13	PLFSHLQNEQWVD
DQ6, DQ7, DQ2, DQ8	DLVLYNNADGDFAIVK
DR2, DR5	FLMAHYNRVPALPFPGDPRP
	LWVLRVPSTMVWRPDIVLEN
	IVVNAVVVLNVSLRSP
	VRKVFLRLLPQLLRMHVRPL
DR2, DR5, DR3	NRVPALPFPGDPRPYLPSPD
DR3	PPAIFRSACSISVTYFPFDW
	FPFDWQNCSLIFQSQTYSTN
	GQTIEWIFIDPEAFTENGEW
DQ2, DQ7, DQ3t12	IHIPSEKIWRPDLVLY
DR3, DR11	IWRPDVVLYNNADGDFAIVKFTKVLLDYTGHITWTPPAIFKSYCEIIVTHFPFDEQNC
DQ8, DQ2, DQ6, DQ7, DQ3t12, DQ3.33	PDTPYLDITYHFVMQRL
DQ8	AIFKSYCEIIVTHFPFD
DQ8, DQ5, DQ7, DQ2, DQ3t12	EVNQIVTTNVRLKQQW
DQ8, DQ5, DQ6, DQ7, DQ3.33, DQ3t12	EDHRQVVEVTVGLQLI
DQ8, DQ6, DQ7, DQ3.33, DQ3t12	WNPDDYGGVKKIHIPS
DQ8, DQ6, DQ7, DQ2, DQ3t12	RGWKHSVTYSCCPDTPY
DQ8, DQ7, DQ3t12	HFPFDEQNCSMKLGTWT
DQ8, DQ6, DQ3.33	LKQQWVDYNLKWNPDD
DQ2, DQ5, DQ6	FMESGEWVIKESRGWKH
DQ2, DQ5, DQ7, DQ3t12	GLQLIQLINVDEVNQI
DQ2, DQ6	SEHETRLVAKLFKDYS
DQ2, DQ6, DQ5, DQ7, DQ3.33	LGTWTYDGSVVAINPES
DQ2, DQ6, DQ7, DQ3.33	QYTGHITWTPPAIFKS
DQ2, DQ7, DQ6, DQ5, DQ8, DQ3t12, DQ3.33	FKDYSSVVRPVEDHRQ
DQ2, DQ8, DQ5	INPESDQPDLSNFMESG

Data were retrieved by querying for all human data against all AChRs. T- and B-cell Assay data were downloaded separately into Excel format and used to filter on the column “h_mhc_types_present.” The Allele Finder on the homepage was used to decipher serotype from complex alleles. Prolif: T-cell proliferation assay; exac: disease exacerbation assays; IFNg or IL-2: cytokine release assays.

**Table 6:** AChR-specific monoclonal antibodies.

MAb name	Cross-react?	Sequence
WF5	ND	VDEVNQIVET (67–76)
42, 176, 177, 203	ND	RLRQQWIDVRLRWNPADYGG (79–98)
28, 37, 42, 176, 177, 203	Y	RWNPADYGGIK (90–100)
6, 22, 47, 50, 198	ND	WNPAD
28, 35, 37, 42, 111, 203	ND	WNPADYGGIK
6, 198, 210	ND	WNPADYGGIKKIRLPSDD
WF6	ND	NNADGDFAIVHMTKLLLDYT (118–137)
WF6	ND	LDYTGKIMWTPPAIFKSYCE (134–153)
FK1	ND	WTPPAIFKSYCEIIVTHFPF
258	ND	YCEIIVTHFPFDQQNCT
5D9 (B-2-ADNR)	Y**	VRTVESGECTIQFFSNAAVTFGTAI (169–193)
236	ND	ESDRP
WF6	ND	PESDRPDLSTFMESGEWVMK (184–203)
A7	ND	VSISPESDRPDLSTF
WF6	ND	SISPESDRPDLSTFMESGEW
WF6	ND	NCTMKLGIWTYDGTKVSISP (165–184)
236, 237	ND	DGTKVSIS
A14	ND	LSTFMESGEWVMK (191–203)
A13	ND	SPESDRPDLSTFMESGE
4B	ND	GWKHWV
9B	ND	KHWVYY
31I	Y	SGEWVMKDYRGWKHWVYYTCCPDTPYLDITYH
383C	ND	WVYYTCCPDTPYL
370A	ND	WVYYTCCPDTPYLDITYHF
WF6	ND	YRGWKHWVYYTCCPDTPYLD (205–224)
WF6	ND	WKHWVYYTCCPDTPYLDITY
3B	ND	YYTCCP
255	ND	DSGEKM
254, 255	ND	LPTDSGEK (259–266)
*α*304–322/1, *α*304–322/2	ND	RKIFIDTIPN
*α*304–322/3	ND	STHTMPQWVRKIFIDTIPN (328–346)
10	ND	PSPDSKPT
117	ND	DSKPTIISRAN
169	ND	VTTPSPDSKPTIISRANDEYFIRKPAGDFVCPVDNAR (360–396)
110, 111, 112, 114, 118, 120, 123, 151	Y	NDEYFIRK
124, 148	Y	EYFIRK
5, 19, 142	ND	IDISDISGKQVTGEVIFQT (370–388)
3, 5	ND	SDISGKQVT
142	ND	GEVIFQ
142	ND	GKQVTGE (377–383)
149	ND	IFAD
13	ND	KIFADDID
187	ND	ASKEKQENKIFADDIDISD (356–374)
149, 187	ND	NKIFADDI
A3	ND	NKIFADDIDISDISGKQVTGEVIFQTPLIKNPDVKSAIEGVKYIAEHM
61	Y	AIEGVKYI
152, 153, 157, 164	ND	DVKSAIEGVKYIAEH
8	ND	PDVKSAIEGV
155	Y	DVKSAIEG
10, 147	ND	VIFQTPLIKNP
8, 147, 153, 155, 164	ND	VIFQTPLIKNPDVKSAIEG (384–402)
147	ND	PLIKNPDVKSAI
152, 153, 155, 157, 164	Y	KSAIEGVK
61	ND	SAIEGVKYIAEHMKSDEESSN
147	ND	NPDVKSAI
8	ND	LIKNPDV
61	ND	IGTVSVFAGRLIELSQEG (444–461)
252	ND	GRLIELSQEG
7	Y	RHGLKR (398–403)
154, 165, 168	ND	EYILKKPRS (380–388)
125	ND	QYVAMVADRLFLY (453–465)
145	ND	TSDIDI (408–413)
111, 148	ND	VDNARVAVQPERLFSEMKWHLNGLTQPVTLPQDLKEAVE (392–430)
6, 22, 35, 42, 47, 198	ND	N92, D95 (nonlinear)
637	ND	21–52, 80–101 (nonlinear)

These data were generated by performing an advanced B cell Search from the drop down menu. The epitope source antigen was selected, along with Qualitative Measurement equal to “positive” and Assayed Antibody Purification Status equal to “monoclonal.” This same search menu enables the user to search by mAb name if data captured for a specific monoclonal is desired. “Y”: the mAb was shown to cross-react with a human AChR; “ND”: not determined by assays present in the IEDB. **Cross-reacts with *β*_2_ adrenergic receptor. Sequence positions given in parentheses were those provided by the authors.

**Table 7:** Epitopes associated with immunomodulation.

Myasthenogenic epitopes	Antigen	Response
** **DTPYLDITYHFVMQRLPL (240–257)	Human AChR	T-cell
** **VIVELIPSTSSAV (304–316)	Human AChR	T-cell
** **DGDFAIVKFTKVLLDYTGHI (117–136)	Rat AChR	T-cell Assay
** ****KSYCEIIVTHFPFDEQNCSMKLG (125–147)	HumanAChR	T-cell Assay
** ***LGIWTYDGTKVSISPES (170–186)	*T. californica *AChR	T-cell Assay
** *****YAIVHMTKLLLDYTGKI (110–116)	*T. californica *AChR	T-cell Assay

Tolerogenic/Therapeutic epitopes		

** **FEQAVEWLVKESRK (722–735)	*H. influenzae* DNA gyrase subunit B	T-cell Assay
** **NSQPEILERTRAELD (89–103)	Mouse IA-beta subunit	T-cell Assay
** ***LGIWTYDGTKVSISPES (170–186)	*T. californica* AChR	T-cell Assay
** **KSYCEIIVTHFPFDQQNCTMKLGI (149–172)	*T. californica *AChR	T-cell Assay
** **TYDGTKVSISPESDRPDLST (174–193)	*T. californica *AChR	T-cell Assay
** **KGNNVHGFQKQIIRSFY (analog of 124–140 region)	Synthetic analog	T-cell Assay
** **RGWKHSVTYSCCPDTPY (227–243)	Human AChR	T-cell Assay
** **ISQAVHAAHAEINEAGR (323–339)	Chicken OVA	T-cell Assay
** **QKSQRSQAENPV (analog of 74–85 region)	Synthetic analog	T-cell Assay
** *****YAIVHMTKLLLDYTGKI (110–116)	*T. californica *AChR	T and B cell Assaies
** **VIVKLIPSTSSAVDTPYLDITYHFVAQRLPL	Synthetic analog	T and B cell Assaies
** ** (analog of 259–271 and 195–21 regions)		
** **GGIKKIRLPSDDVWLPD (97–113)	*T. californica *AChR	B cell Assay
** **ISGKQVTGEVIFQTPLIK (375–392)	*T. californica *AChR	B cell Assay
** **DLKLRRSSSVGYIS (375–388)	*T. californica *AChR	B cell Assay
** **ISGKPGPPPMGFHSPLIK (396–413)	Human AChR	B cell Assay
** **KGNNVHGFQKQIIRSFY (analog of 124–140 region)	Synthetic analog	B cell Assay
** **NIHPKAPIWGIITSNF (analog of 61–76 MIR region)	Synthetic analog	B cell Assay
** **DYTGKIMWTPPAIFKS (135–150)	*T. californica *AChR	T-cell Assay

Data were derived by querying the database for EAMG. Positive T-cell and B cell assay data were downloaded using Excel format. For Myasthenogenic peptides the worksheet was filtered for “Administration *in vivo* to cause disease” in the following columns: “1st *In Vivo* Process Type” or “2nd *In Vivo* Process Type” or “Adoptive Transfer *In Vivo* Process Type,” one at a time. For tolerogenic or therapeutic peptides the worksheet was filtered for “Administration *in vivo* to prevent or reduce disease” in the in the same columns. *Asterisk indicates that the peptide was shown to both induced disease and to reduce disease depending upon the sequence given.**This peptide was synthesized with a norleucine substitution at M20. ***Disease enhancement was associated with adoptive transfer of epitope-specific T-cells; pretreatment induced tolerance. It is also possible to search by assay type: treatment assay, tolerance induction, and exacerbation. Typing the exact sequence into the linear peptide field on the Home Page Search Interface will generate results summary table for the investigation of individual records.
